# Nuclear distribution of porphobilinogen deaminase (PBGD) in glioma cells: a regulatory role in cancer transformation?

**DOI:** 10.1038/sj.bjc.6600173

**Published:** 2002-03-18

**Authors:** L Greenbaum, Y Gozlan, D Schwartz, D J Katcoff, Z Malik

**Affiliations:** Faculty of Life Sciences, Bar-Ilan University, Ramat-Gan 52900 Israel

**Keywords:** glioma c6, protoporphyrin, porphobilinogen deaminase, differentiation

## Abstract

Recently, considerable interest has been directed to red-fluorescence photodiagnosis of brain and other tumours during surgery using the protoporphyrin IX natural precursor, 5-aminolaevulinic acid. In the present study we focused on the role of the rate-limiting enzyme porphobilinogen deaminase in glioma C6 cell activity, differentiation and sub-cellular distribution. Over-expression of the human housekeeping porphobilinogen deaminase in the glioma cells, using the housekeeping-porphobilinogen deaminase plasmid, induced a G1 cell cycle attenuation accompanied by increases in enzyme activity and c6 differentiation toward astrocytes. Visualisation of subcellular localisation of the porphobilinogen deaminase using the independent techniques of fluorescence immuno-staining with specific anti-human porphobilinogen deaminase antibodies and cellular expression of porphobilinogen deaminase fused to green fluorescent protein, revealed (unexpectedly) a major fraction of porphobilinogen deaminase in the nucleus and only a minor fraction in the cytoplasm. Both C and N terminals of porphobilinogen deaminase fused to green fluorescent protein revealed a major fraction of the newly synthesized fused porphobilinogen deaminase in the nucleus. Furthermore, newborn rat brain cells grown in a primary culture showed the same localisation pattern of porphobilinogen deaminase in the nuclei. Stimulation of C6 glioma cell differentiation by butyrate induced a marked decrease in porphobilinogen deaminase both in the nucleus and in the cytoplasm as determined by Western blotting and fluorescence immuno-localisation. These findings suggest a possible dual role for housekeeping porphobilinogen deaminase in fast dividing glioma cells, one related to the porphyrin synthesis pathway and another coupled to nuclear function, which might be linked to tumorigenesis.

*British Journal of Cancer* (2002) **86**, 1006–1011. DOI: 10.1038/sj/bjc/6600173 www.bjcancer.com

© 2002 Cancer Research UK

## 

Malignant gliomas accumulate fluorescing protoporphyrin intra-cellularly after exposure to the natural precursor 5-aminolevulinic acid (ALA), similar to a variety of tumours and leukemic cells (Malik and Lugaci, 1987; Malik *et al*, 1989, 1996, 1998; Peng *et al*, 1997a,b; Stummer *et al*, 1998a,b; Kennedy *et al*, 1990; Kennedy and Pottier, 1992). This phenomenon has been exploited for complete removal of malignant gliomas (Stummer *et al*, 1998a,b). The intraoperative identification of tumor tissue was performed by application of ALA prior to surgery and subsequent observation with the operating microscope with excitation using a violet-blue (375–440 nm) xenon light, a 455-nm long-pass filter and verification by analysis of fluorescence spectra. Protoporphyrin-fluorescence guided-resection resulted in residual tumour removal, which improved patient prognosis (Stummer *et al*, 1998a,b).

The biosynthetic pathway of protoporphyrin and heme (Kennedy *et al*, 1990; Peng *et al*, 1997a,b) consists of several enzymatic steps: (1) Glycine + Succ.CoA → 5-aminolevulinic acid (ALA) → porphobilinogen (PBG); (2) 4PBG → uroporphyrinogen → coproporphyrinogen → protoporphyrin + Fe^++^ → heme. All heme synthesis enzymes are exclusively localized in the cytoplasm except for the first enzyme (ALA-synthase) and the two last enzymes (protoporphyrinogen oxidase and ferrochelatase) that are known to function as mitochondrial enzymes. Jorgensen *et al* (2000) recently showed in paraffin embedded specimens that porphobilinogen deaminase (PBGD), the most studied enzyme in heme synthesis, is localized in the cytoplasm of rat kidney. Supplementation of ALA circumvents the rate-limiting enzyme ALA synthase (Malik and Lugaci, 1987) and enhances the accumulation of protoporphyrin if the activity of the PBGD, at the second check point, is sufficiently high. One possible rationale for the specificity of protoporphyrin accumulation in rapidly dividing cancer cells is that their enhanced metabolism demands additional heme, which is needed for increased aerobic ATP supply connected to the energy demands of the tumour. Malignant transformation of various cell lines by retrograde transforming viruses and other means leads to increased PBGD activity (Rasetti *et al*, 1963; Kondo *et al*, 1993). Similarly, the malignant lymphoproliferative diseases of chronic lymphocytic leukaemia and lymphoma were found to have higher PBGD activity in their peripheral lymphocytes than normal control subjects (Leibovici *et al*, 1988). However, it is unclear whether the increase in PBGD activity was caused by malignant transformation or whether it was dependent on the increased growth rate.

The purpose of the present study is to elucidate the role of PBGD in C6 glioma cells, focusing on the correlation between cell proliferation and subcellular localisation of the protein. Our findings indicate possible nuclear functions of PBGD, which may be involved in the proliferation and differentiation of glioma cells apart from the basic cytoplasmic enzymatic activity of PBGD in porphyrin synthesis.

## MATERIALS AND METHODS

### Cell culture

Rat C6 glioma cells were grown in a DMEM medium (Biological Industries, Kibbutz Beit-Haemek, Israel), supplemented with 10% foetal calf serum and antibiotics, on tissue culture plates (Corning, Staffordshire, UK) and incubated at 37°C in a humidified atmosphere with 8% CO_2_. The cells were recultured twice a week, using Trypsin-EDTA for detachment.

### Primary newborn brain cell culture

Newborn rats brains were removed under sterile conditions, washed with PBS followed by trypsinisation under stirring for 10 min at room temperature. The supernatant was removed into a 50 cc test tube containing foetal calf serum (Biological Industries, Beit Haemek, Israel). After 10 min of centrifugation at 1200 r.p.m., the pellet was resuspended in DMEM supplemented with foetal calf serum, L-glutamine and antibiotic. The primary cell culture was plated for 20 h at the same conditions as the C6 cells.

### Preparation of human PBGD cDNA expression vectors

The coding region of the erythrocyte PBGD cDNA was amplified from a human spleen cDNA library in bacteriophage λGT10 (a gift from P Sankhavarar, Yale University) using 5′-GAAGATCTATGAGAGTGATTCGCGTGGGTACC-3′ forward primer and 5′-GGAATTCTTAATGGGCATCGTTAAGCTGCCG-3′ reverse primer. The PCR product was used as a template for a PCR reaction using the forward primer: 5′-GAAGATCTATGTCTGGTAACGGCAATGCGGCTGCAACGGCGGAAGAAAACAGCCCAAAGATGAGAGTGATTCGCGTGGGT-3′ and the reverse primer 5′-GGAATTCTTAATGGGCATCGTTAAGCTGCCG-3′. The PCR product was inserted into the pLY-3 plasmid (pEGFP-C1 plasmid [Clontech, Palo Alto, CA, USA]) we created using *Sma*I and *Eco*47III restriction enzymes that lacked the green fluorescent protein (GFP) coding region, yielding a new plasmid called pHK-PBGD plasmid, which expresses the housekeeping PBGD (HK-PBGD) in mammalian cells.

The PBGD coding region was cut out from pHK-PBGD using *Bgl*II and *Eco*RI restriction enzymes and it was ligated to pEGFP-C1 or pEGFP-N1 (Clontech, Palo Alto, CA, USA) in frame to the C- or N-terminal of GFP. The ligation product expressed EGFP fused to HK-PBGD. Constructs were verified by sequencing (Weizmann Institute Biological Services, Rehovot, Israel).

### Cell transfection with mitochondrial-GFP and PpIX localisation

For transient transfection, C6 cells were incubated with serum-free medium for 40 min followed by 6 h incubation with transfection solution containing 0.65 μg ml^−1^ of mitochondrial GFP plasmid (pEGFP-Mito) (Clontech, Palo Alto, CA, USA) (encoding the GFP which translocalised specifically into mitochondria) and 6.7 mg ml^−1^ lipofectamine reagent (GIBCO-BRL, NY, USA) in DMEM. Following transfection, the cells were transferred to a rich medium containing serum and antibiotic. Six hours before detection of PpIX localisation in the transfected cells, the cells were incubated with serum-free medium containing 0.6 mM amino-levulinic acid (Sigma, St. Louis, USA).

### Stable and transient cell transfection

The pHK-PBGD plasmid was transfected into C6 cells, using lipofectamine reagent (GIBCO-BRL, NY, USA) according to the manufacturer's protocol. For stable transfection, a selective medium containing 850 μg ml^−1^ neomycin, also known as geneticin 418 (GIBCO-BRL, NY, USA) was added to the cells 24 h post-transfection. The surviving neomycin-resistant colonies on each plate were then subcloned by dilution and grown to establish the sub-lines. For selection maintenance, the cells were selectively grown in a medium containing 100 μg ml^−1^ geneticin. For PBGD fused to GFP as a transient transfection, the procedure was exactly as mentioned above, without adding neomycin. The cells were examined 24 h post transfection.

### Western blotting

Proteins were quantified using the Bradford assay (Bio-Rad, CA, USA) and resolved in a 12% polyacrylamide gel. Thereafter, proteins were transferred onto nitrocellulose membranes using a semi-dry transfer apparatus (Bio-Rad, CA, USA). After blocking of the membranes with 5% skim milk and 0.06% Tween-20 in PBS, membranes were incubated with primary PBGD antibody (a generous gift from HemeBiotech, Sweden), and goat anti-rabbit secondary antibody (Jackson Immuno-Research, Pennsylvania, PA, USA) in the same solution. Immuno-reactive proteins were visualised with an enhanced chemiluminescence detection kit (Amersham, Little Chalfont, UK) used as recommended by the manufacturer.

### PBGD, glial fibrilary acid protein (GFAP) and vimentin immunolabelling, and GFP–PBGD detection

Cells were seeded in eight-well slide chambers. Twenty-four hours later, cells were fixed using 4% paraformaldehyde and subsequently treated with 0.5% Triton X-100 for 30 min. Blocking was carried out with 6% skim milk, 3% BSA<and 0.2% Tween-20 in 100% FCS. Cells were then exposed to one of the next primary antibodies overnight at 4°C: Rabbit polyclonal anti-PBGD antibody (HemeBiotech, Stockholm, Sweden), rabbit polyclonal anti-GFAP antibody (Dakopatts, Glostrup, Denmark) or mouse monoclonal anti-vimentin antibody (Dakopatts, Glostrup, Denmark) Reacting rhodamine-conjugated anti-rabbit and anti-mouse antibodies (Jackson Immuno-Research, Pennsylvania, PA, USA). The cells were visualized with fluorescence microscopy (Olympus AX70).

### Fluorescence microscopy

Fluorescence microscopy was performed using an Olympus AX70 microscope attached to a SpectraCube™ SD-200 spectral imaging system (Applied Spectral Imaging, Migdal HaEmek, Israel) operated as described by Malik *et al* (1996). Photography was carried out with an ×60 objective. For Rhodamine the excitation was at 570 nm and an image acquired at 610–700 nm; for GFP, excitation was at 420 nm and emission at 515–630 nm.

### PBGD enzymatic activity assay

PBGD is assayed by determining the absorbance of uroporphyrin that is formed by light-induced oxidation of uroporphyrinogen, which is the immediate product of the enzymatic deamination. 10^6^ C6 cells were detached from plates 24 h after plating, and resuspended in PBS without Ca^2+^/Mg^2+^. After centrifugation, the pellet was resuspended in 1 ml 50 mM tris (pH 8.2). 250 μl lysate were incubated with an equivalent amount of Triton-X-100 contained tris buffer and 100 μl of 0.5 mM porphobilinogen (PBG) (Porphyrin Products, UT, USA) for 1 h at 37°C with shaking. The reaction was stopped by addition of 10% TCA under exposure to ambient room light at room temperature for 10 min. After 10 min centrifugation (3300 r.p.m.), the supernatant was collected to the spectrofluorometer (ex. 409 nm, em. 595 nm) (Spectronic Instruments, Leeds, UK). PBGD specific activity is expressed as pmol uroporphyrin formed per mg protein per 1 h units.

### Nucleus–cytoplasm fractionation

C6 cells were harvested and resuspended in Tris-HCl buffer supplemented with EDTA, EGTA and the antiproteases leupeptine, aprotinine, PMSF and DTT (Sigma, St. Louis, USA). For nucleus–cytoplasm fractionation, cells were homogenised on ice followed by centrifugation at 2500 r.p.m. for 10 min (twice), and the nuclear fraction was resuspended in the above buffer. For Western blotting of each fraction, Triton-X-100 was added and the samples were immersed in nitrogen liquid and thawed at 37°C intermittently, followed by centrifugation at 14 000 r.p.m. for 20 min.

## RESULTS

**Figure 1:**
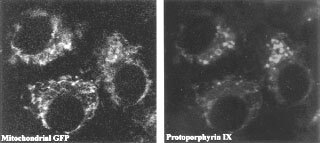
Co-localisation of PpIX and mitochondrial-GFP in C6 glioma cells. The cells were transiently transfected with mitochondrial-GFP plasmid pEGFP-Mito and incubated for 6 h with 0.6 mM ALA. The green fluorescence of GFP and the PpIX localisation in the cells were shown by green and red filters as detected by fluorescence microscopy (left and right panels, respectively) with an ×60 objective.

The capacity of C6 glioma cells to synthesize protoporphyrin following ALA supplementation is revealed in Figure 1

; protoporphyrin accumulated in the mitochondria and cytosol. The mitochondria were identified by mitochondrial-targeted GFP (the GFP contains a mitochondrial targeting sequence directing the protein to translocalize into mitochondria), which partially co-localizes with the red protoporphyrin fluorescence.

**Figure 2:**
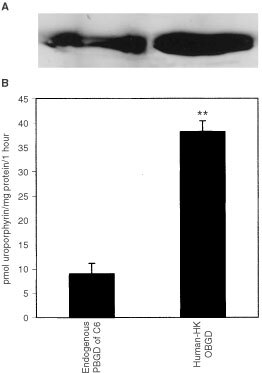
Over-expression of PBGD in C6 glioma cells. (**A**) Western blotting of HK-PBGD. (**B**) The enzymatic activity of PBGD in control C6 and over-expressing cells (**P<0.005, as measured from five different experiments).

**Figure 3:**
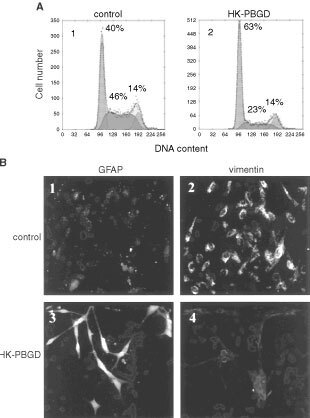
The effect of PBGD over-expression on the cell cycle (**A**) and GFAP/vimentin intermediate filaments phenotype (**B**) in C6 glioma cells. A1- cell cycle of C6 control cells, A2- C6 cells over-expressing the housekeeping PBGD. The GFAP/vimentin immuno-staining is shown in **B**. B1, B3 – immuno-staining with rabbit polyclonal anti-GFAP antibody and B2, B4 – mouse monoclonal anti-vimentin antibody immuno-staining as detected by fluorescence microscopy.

In order to affirm the postulated association between PBGD activity, C6 cycle and tumour outgrowth, we over-expressed housekeeping PBGD in C6 glioma cells by stable transfection with the pHK-PBGD plasmid. Western blotting of C6 endogenous PBGD as well as of stable transfected cells over-expressing PBGD, shows an intense PBGD expression in the transfected cells (Figure 2A

). As an outcome of over-expression, PBGD activity was increased fourfold from 9 to 38 pmol uroporphyrin mg^−1^ protein per 1 h (Figure 2B). The increased activity in PBGD was accompanied by attenuation at the G1 phase of the cell cycle as revealed by flow cytometry (Figure 3A

). The G1 value of the PBGD transfected cells was 63% (Figure 3, A2) in comparison to 40% of control C6 glioma cells (Figure 3, A1) or those transfected with a control pEGFP plasmid (data not shown). The increase in G1 population of the PBGD over-expressing cells was the result of a marked reduction in the S phase fractions. No significant S/G2 transitions were revealed in this experiment. The accumulation of C6 cells in the G1 phase may represent a differentiation process leading to differentiated astrocytes. The transition from a vimentin producing phenotype of highly proliferating control cells to GFAP intermediate filament phenotype in the PBGD over-expressing cells (Figure 3B) indicates a differentiation process into astrocytes (Toda *et al*, 1994). An intense vimentin and basal GFAP staining was observed in non-transfected cells (Figure 3, B2 and B1, respectively), while GFAP immunofluorescence dramatically increased upon PBGD over-expression and the vimentin was down regulated (Figure 3, B3 and B4, respectively). The immunofluorescence of both vimentin and GFAP was revealed in the perinuclear region and in filametous structures, and the intense satellite-like structure of GFAP is known as typical to astrocytes.

**Figure 4:**
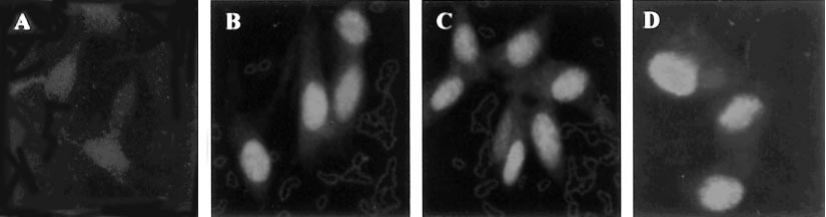
Nuclear localisation of PBGD in C6 cells detected by fluorescence immuno-labelling. (**A**) is a control in which anti-PBGD antibody was withdrawn from the samples, (**B**) is the endogenous PBGD, (**C**) is the over-expressing human housekeeping PBGD and (**D**) is the endogenous PBGD immuno-labelling of newborn primary brain cells. The nuclear *vs* cytoplasmic distribution of PBGD was a typical result in all experiments, which in most of the C6 cells was highly pronounced.

These results suggested that we attempt to visualise PBGD at the sub-cellular level. To that end we used fluorescence immuno- labelling using specific human PBGD antibodies and cellular expression of the fusion protein GFP-PBGD. These two independent techniques allowed visualisation of the subcellular localisation of endogenous as well as over-expressed PBGD in the C6 glioma cells (Figures 4

and 5

**Figure 5:**
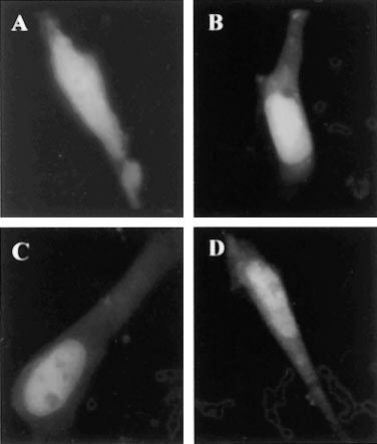
Over-expression of human housekeeping PBGD fused to GFP. Green fluorescence of C6 cells expressing GFP fused to the N-terminal (**C**), or to the C-terminal (**D**), 24 h post-transfection. The negative and positive controls for nuclear GFP localisation were cells expressing wild type GFP-N1 plasmid revealing diffused fluorescence (**A**) and cells expressing the SV-40 NLS GFP plasmid showing nuclear localisation (**B**).

). As was expected, a fraction of the PBGD immuno-fluorescence complex was found in the cytoplasm (Figure 4B,C). However, as can be seen in Figure 4B,C, the major fraction of the PBGD immuno-complex was detected in the nucleus. Nuclear PBGD localisation was a typical result in over 90% of the C6 cells in all experiments. Furthermore, newborn rat brain cells grown for 20 h as a primary culture showed the same nuclear localisation of PBGD (Figure 4D).

Over-expression of PBGD fused to GFP (at either the C or N terminals) in the glioma cells revealed that the major fraction of the newly synthesised fused proteins is localised in the nucleus (Figure 5C,D). This confirmed the results found using immuno-labelling. Control cells transfected with wild-type GFP alone showed diffused distribution of the label throughout the cell (Figure 5A), while control NLS-GFP (Figure 5B) was localised specifically in the nucleus.

**Figure 6:**
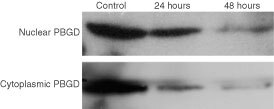
Western blot detection of PBGD localisation in C6 glioma cells during differentiation. PBGD in nuclear (upper panel) and cytoplasmic (lower panel) fractions was detected after 24 and 48 h of 2.5 mM butyric-acid treatment.

**Figure 7:**
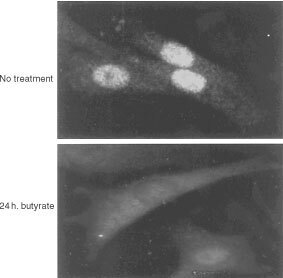
PBGD immuno-localisation in C6 glioma cells during differentiation. Intact cells are shown in the upper panel, while cells treated for 24 h with 2.5 mM butyric-acid are shown in the lower panel. This result, of pronounced nuclear PBGD localisation in undifferentiated cells and a reduced nuclear and cytoplasmic distribution, was found in all experiments that were carried out.

These results pointed to a possible dual role for PBGD in rapidly dividing cells, one that is related to the porphyrin synthesis pathway and another coupled to nuclear function. Therefore, the C6 glioma cells were stimulated by butyrate, to induce differentiation, and the nuclear *vs* cytoplasmic distribution of PBGD was analysed using Western blotting (Figure 6

). In the control uninduced glioma cells, the endogenous PBGD was comparatively high both in the isolated nuclei and the cytoplasmic fraction. After 24 h of butyrate induction, PBGD decreased both in the nuclear fraction as well as in the cytoplasmic fraction. This trend was further seen after 48 h, at which time the total PBGD was reduced and it was distributed between the nucleus and cytosol. Fluorescence immuno-localisation of the PBGD in the butyrate-stimulated cells revealed a marked reduction in the nucleus and the cytoplasm (Figure 7

). This result of pronounced nuclear PBGD localisation in the undifferentiated cells, and reduced nuclear and cytoplasmic distribution was found in all experiments that were carried out.

## DISCUSSION

The regulatory role of porphobilinogen deaminase in PpIX synthesis following ALA administration is considered the rate-limiting determinant and thus porphobilinogen deaminase is the key enzyme controlling photodiagnosis and photodynamic therapy (Gibson *et al*, 1998). Transient transfection of cells with the cDNA of PBGD was successful in elevating enzyme activity in both the human mammary tumour cell line MCF-7 and the human mesothelioma cell line, H-MESO-1, but this did not result in a comparable difference in the levels of PpIX (Hilf *et al*, 1999). PBGD has always been considered exclusively as a cytoplasmic enzyme of the heme biosynthesis pathway (Sassa, 1990). Jorgensen *et al* (2000) have shown immuno-histochemical localisation of PBGD in various rat tissues, in which PBGD was unevenly distributed among various cell types in a given tissue. However, nuclear localisation was not indicated. Interestingly, our present results reveal that a major fraction of the PBGD is localised specifically to the nucleus, while a relatively minor fraction is found in the cytoplasm. Since our study discusses an additional, novel, function of a known protein, which derived from its sub-cellular localisation, we confirmed the localisation findings also by immuno-labelling and GFP-tagging. The independent localisation methods, both demonstrating a nuclear fraction of PBGD, strongly indicate a nuclear-specific transport and a possible nuclear function of this protein. Nuclear localisation was seen for both the endogenous PBGD and the over-expressed human-housekeeping PBGD in glioma cells. Tagging the GFP to either the N or C termini showed the same results, which means that the PBGD nuclear localisation signal is not located in the ends of the protein.

It is well documented that nuclear localisation signals often contain lysine–arginine inner repeats separated by non-specific residues (Martelli *et al*, 1999). Sequence analysis of the human PBGD protein revealed RR X5 RK X17 RK repeats between residues 149 and 180, which may fulfil the requirements for a nuclear localisation signal. Nuclear localisation of PBGD is consistent with its involvement in G1 attenuation. The enzymatic activity of PBGD in the over-expressing cells was increased fourfold, and in addition, G1 cell cycle attenuation was revealed. It is well known that cells that pass the G1 phase are committed to complete cell division. Regulation of the G1 phase in a cell cycle is complex and involves many different families of proteins (Donjerkovic and Scott, 2000), some of which are cell-type specific and thus related to cell differentiation (Coffman and Studzinski, 1999). Our model system, based on a rapidly proliferating rat C6 glioma cell line which has oligodendrocytic, astrocytic, and neuronal properties (Parker *et al*, 1980; Segovia *et al*, 1994), is widely used to study differentiation processes. Astrocyte-like differentiation involves a shift from vimentin synthesis toward glial fibrillary acidic protein (GFAP) production (Roymans *et al*, 2000) followed by morphological changes. Our present results show typical morphological changes to an astrocyte-like phenotype revealing perinuclear GFAP satellite-like formation in the PBGD transfected cells. This result correlates well with a reduction in vimentin synthesis. Since these findings are joined both to G1 attenuation and to C6 differentiation in PBGD-transfected cells, we assume that PBGD plays an additional role in cell differentiation or in other cellular processes. Piatigorsky provided evidence for multiple protein functions for a single protein involved in different cellular mechanisms (Piatigorsky, 1992). The δ-aminolevulinic acid dehydratase, which is also an enzyme that participates in the heme biosynthesis pathway, has been shown to play an additional role as inhibitor of the 26S proteasome (Guo *et al*, 1994).

Schoenfeld showed that increased activity of PBGD is strongly correlated with an increased growth rate in both non-malignant and malignant cell lines (Schoenfeld *et al*, 1988). The power of elevated activity of PBGD to produce PpIX was clinically demonstrated for malignant gliomas (Stummer *et al*, 1998b); oral cavity cancer (Leunig *et al*, 2000a); colonic and gastrointestinal dysplasia (Stepp *et al*, 1998); peritoneal endometriosis (Hillemanns *et al*, 2000); laryngeal neoplasms (Leunig *et al*, 2000b); malignant lesions of the oesophagus (Hinnen *et al*, 1998); lower urinary tract tumours (Kriegmair *et al*, 1999); bladder cancer (Kriegmair *et al*, 1996); malignant mucosa in head and neck cancer (Betz *et al*, 1999); Barrett's oesophagus and adenocarcinoma (Stepp *et al*, 1998); basal and squamous cell carcinoma (Orenstein *et al*, 1995, 1996, 1997; Malik *et al*, 1998) and coetaneous lymphoma (Orenstein *et al*, 2000).

A direct correlation between the intracellular accumulation of PpIX and malignancy could not always be found, as in the case of Barrett's esophagus and adenocarcinoma. However, higher heme biosynthetic enzyme activities (i.e. PBGD) and lower PpIX precursor concentrations were found in squamous carcinoma (Hinnen *et al*, 2000).

Consistent with the increased activity of PBGD in proliferating cells, our results show decreased nuclear and cytoplasmic PBGD levels in differentiating cells. Additionally, Lindberg showed recently that mutant mice lacking the PBGD gene exhibit a marked decrease in large-caliber axons and manifest general motor neuropathy (Lindberg *et al*, 1999). This reduced activity of PBGD correlates with an additional role for PBGD in differentiation.

Photodiagnosis and protoporphyrin IX-dependent phototherapy are highly successful techniques with a wide range of clinical therapeutic application (Peng *et al*, 1997a,b). On the basis of the present findings, we may conclude that the intracellular changes in PBGD levels is intimately connected to differentiation. The markedly increased capacity of tumours to produce and accumulate PpIX, which is directly associated with elevated PBGD activity, possibly reflects an intrinsic virtue of the tumour metabolic capacity.
